# Audit of Community-Based Relapse-Prevention Planning Following Successful Alcohol Detox on Referral to an Inpatient Detox Unit in the East Midlands

**DOI:** 10.1192/bjo.2026.11736

**Published:** 2026-06-30

**Authors:** Lai-Ting Cheung, Sue Tan, Olawale Lagundoye

**Affiliations:** Nottinghamshire Healthcare NHS Foundation Trust, Nottingham, United Kingdom

## Abstract

**Aims:**

Our patients are referred locally and out of area but it was noted relapse prevention plans were not documented in sufficient detail. We wanted to improve relapse prevention and post-discharge care. Our aims were to:-

- Audit details of alcohol relapse prevention work started pre-admission and post-discharge plans
- Assess if alcohol relapse prevention work was considered from a bio-psycho-social perspective.

**Methods:**

Retrospective audit of electronic referral forms for patients admitted between 3^rd^ November 2025-28^th^ November 2025. We reviewed referrer details, preparatory work, relapse prevention plans, relapse prevention medication (RPM), psychological support, mental health and social situation.

**Results:**

22 patients were admitted between 3^rd^ November 2025-28^th^ November 2025. 19 (86.36%) were out of area and 3 (13.64%) were from the local service.

Information on preparatory work was not available in 16 of the 22 referrals (72.73%). There were written plans in 15 of the 22 referrals (68.18%). Of the 7 going to rehab, 2 did not have a written plan (28.57%).

RPM was mentioned in 18 of the 22 referrals (81.82%). 12 (54.55%) had requested acamprosate. 2 (9.09%) had requested disulfiram and 1 (4.55%) had requested naltrexone. Two or more options were considered in 2 referrals (9.09%) and 1 was undecided (4.55%).

13 of the 22 referrals (59.09%) mentioned psychological support. Relapse prevention plans included 1:1 sessions, group work, rehab and skills training. Groups included support groups, formal recovery groups, local groups and community groups. Skills included workshops, psychology and employment support. One was homeless and they had a housing plan (100%).

Mental health was mentioned in 19 of the 22 referrals (86.36%). Of the 19, 4 (21.05%) were known to a local mental health team (LMHT) and 1 (5.26%) mentioned plans to refer to LMHT. 13 (68.42%) did not mention plans to refer to LMHT and 1 (5.26%) had declined a referral. Information on mental health was not available in 2 of the 22 referrals (9.09%).

**Conclusion:**

The majority of patients were out of area, which made it difficult to access clinical notes. Therefore, it is important preparatory work and a robust relapse prevention plan is provided.

RPM was well documented but more information was needed on mental health and pre-admission. Following this, we refined the form to capture more detail on preparatory work and to prompt consideration for any mental health support required.

